# Performance of liquid Amies transport medium for the recovery of *Salmonella* and *Shigella* from stool compared to molecular testing

**DOI:** 10.1128/jcm.00453-25

**Published:** 2025-06-04

**Authors:** Laura Collier, Morgan A. Pence

**Affiliations:** 1 Department of Pathology and Laboratory Medicine, Cook Children's Medical Center 14488, Fort Worth, Texas, USA; Children's Hospital Los Angeles, Los Angeles, California, USA

**Keywords:** gastroenteritis, *Shigella*, *Salmonella*

## LETTER

Cary-Blair has long been the gold standard transport medium for bacterial enteric pathogen testing. Its buffering system prevents overgrowth of commensal microbiota while preserving bacterial stool pathogens. Liquid Amies, alternatively, is a nutrient broth and buffering system that prioritizes the viability of microorganisms during transport. Due to a paucity of data evaluating the performance of alternative media for bacterial pathogens, Cary-Blair has remained the standard. When Cook Children’s Medical Center (CCMC) transitioned from culture to molecular testing using the Verigene Enteric Pathogens (EP; Diasorin, Austin, TX, USA) in 2016, stool ESwabs (liquid Amies; Copan, Murrieta, CA, USA) were validated for off-label use due to their historical use for stool cultures at CCMC and the desire to avoid adding another transport medium/collection device. This retrospective study evaluated the performance of ESwabs for the recovery of stool pathogens compared to molecular testing and relative to prior publications that utilized Cary-Blair.

Between December 2016 and December 2024, specimens collected via ESwab and positive for *Salmonella* and *Shigella* by Verigene EP were reflexively cultured in an attempt to recover isolates for antimicrobial susceptibility testing and serotyping. Samples were taken from bulk stool on the unit and placed in the ESwab prior to transport to the lab. Specimens were stored at room temperature and cultured within 48 hours of collection. In-house stool culture included blood agar, MacConkey agar (MAC), and Hektoen Enteric agar (HEA; Thermo Fisher Scientific, Lenexa, KS, USA). Non-lactose-fermenting colonies on MAC and green or black colonies on HEA were subjected to further testing. *Salmonella* isolates were initially identified by Vitek 2 (bioMerieux, Salt Lake City, UT, USA) and subsequently by MALDI-ToF mass spectrometry (Bruker, Billerica, MA, USA) beginning in February 2017. *Salmonella* isolates were subsequently sent to the Texas Department of State Health Services (TDSHS) laboratory for serotyping, initially performed by pulsed field gel electrophoresis (PFGE; 2016–2019) and subsequently by whole-genome sequencing (WGS; 2020–2024). During the transition from PFGE to WGS (June 2019–July 2020), no serotype data were reported (*n* = 244). Additionally, specimens positive for *Salmonella* by Verigene EP that were negative by in-house culture were sent to the TDSHS laboratory for culture within 1 week, which included an enrichment broth in addition to routine solid media. *Shigella* isolates were initially identified by Vitek 2 but transitioned to a combination of MALDI-ToF MS and Wellcolex *Shigella* latex agglutination (Thermo Fisher Scientific, Waltham, MA, USA) in 2020.

A total of 1,899 specimens were positive for *Salmonella* by Verigene EP, of which 1,795 (94.5%) were recovered in culture. Of the isolates recovered in culture, 1,670 (87.9%) were recovered by in-house culture, with an additional 125 (6.6%) recovered by follow-up culture at the TDSHS laboratory. Three hundred ninety-six specimens were positive for *Shigella* by Verigene EP, with 310 (78.3%) positive by in-house culture. A total of 90 serotypes of *Salmonella* and all four species of *Shigella* were recovered during the study period (Tables 1 and 2).

**TABLE 1:** Serotypes of *Salmonella* isolates recovered in culture at CCMC/TDSHS

Serotype	No. of isolates
Newport	251 (14.0%)
Infantis	204 (11.4%)
Typhimurium	199 (11.1%)
Rubislaw	107 (6.0%)
Javiana	99 (5.5%)
Enteritidis	58 (3.2%)
Mississippi	58 (3.2%)
Norwich	43 (2.4%)
I 9:I,z28:-	42 (2.3%)
Montevideo	41 (2.3%)
Other serotype^*a*^	382 (21.3%)
Serotype not provided (June 2019–July 2020)	244 (13.6%)
Undetermined^*b*^	50 (2.8%)
Serotyping not performed^*c*^	17 (0.9%)
Total	1,795

^*a*^ The 10 most common serotypes are shown; 80 additional serotypes were recovered.

^*b*^ A result of Undetermined indicates (i) a duplicate isolate within 6 months from the same patient (at CCMC or another submitting facility) or (ii) an isolate that is not typeable.

^*c*^ The isolate was not sent to TDSHS due to a laboratory error, so serotyping could not be performed.

**TABLE 2:** *Shigella* isolates recovered in culture at CCMC

Species	No. of isolates
*Shigella sonnei*	240 (77.4%)
*Shigella flexneri*	33 (10.7%)
*Shigella boydii*	3 (1.0%)
*Shigella dysenteriae*	2 (0.6%)
*Shigella* species^*a*^	32 (10.3%)
Total	310

^*a*^ *Shigella* isolates were identified at the genus level by Vitek 2 prior to the implementation of Wellcolex *Shigella* latex agglutination in 2020.

Discordant results were consistent with the higher sensitivity expected for PCR-based methods compared to culture but may have also been due to reduced viability of *Salmonella* following transit to the TDSHS laboratory for culture, as well as molecular cross-reactivity between *Shigella* and enteroinvasive *Escherichia coli*, which was difficult to resolve in culture. Similar rates of recovery using Cary-Blair transport media have been reported, with ranges of 83.8%–88.2% for *Salmonella* and 63.3%–80.6% for *Shigella* (1–3).

Specimens collected via ESwab revealed a consistently high recovery rate of *Salmonella* and *Shigella* in culture and subsequent success in serotype/species identification. Although Cary-Blair has traditionally been used for the recovery of stool pathogens, liquid Amies is a viable option for the transport and preservation of *Salmonella* and *Shigella*.
